# Longitudinal integrated evaluation of metabolic, inflammatory, biochemical, and hormonal biomarkers identifies haptoglobin as a key indicator of reproductive performance in Friesian-Holstein cows with endometritis

**DOI:** 10.14202/vetworld.2026.3282-3294

**Published:** 2026-07-28

**Authors:** Herry Agoes Hermadi, Yeni Dhamayanti, Erma Safitri, Rimayanti Rimayanti, Athhar Manabi Diansyah, Langgeng Priyanto, Herdis Herdis, Anita Hafid, Umi Adiati, Muhammad Muflih Abdul Rahman, Ahmad Alfaruqi Syahrandi Adam, Muhammad Fajar Amrullah

**Affiliations:** 1 Division of Veterinary Reproduction, Faculty of Veterinary Medicine, Universitas Airlangga, Surabaya, East Java 60115, Indonesia.; 2 Research Group for Veterinary Reproductive Biotechnology, Faculty of Veterinary Medicine, Universitas Airlangga, Surabaya, East Java 60115, Indonesia.; 3 Division of Veterinary Anatomy, Faculty of Veterinary Medicine, Universitas Airlangga, Surabaya, East Java 60115, Indonesia.; 4 Research Group for Advances in Veterinary Anatomy and Developmental Biology, Faculty of Veterinary Medicine, Universitas Airlangga, Surabaya, East Java 60115, Indonesia.; 5 Department of Animal Production, Faculty of Animal Science, Hasanuddin University, Makassar, South Sulawesi 90245, Indonesia.; 6 Department of Animal Science, Faculty of Agriculture, Universitas Sriwijaya, Palembang 30128, South Sumatera, Indonesia.; 7 Research Center for Animal Husbandry, National Research and Innovation Agency, Cibinong Science Center, West Java 16911, Indonesia.; 8 College of Animal Science and Technology, Department of Animal Genetics Breeding and Reproduction, China Agricultural University, 100193, China.; 9 Graduate Program of Animal Biomedical Science, School of Veterinary Medicine and Biomedical Science, IPB University, Bogor 16680, Indonesia.; 10 Research Center for Applied Zoology, National Research and Innovation Agency, Cibinong Science Center, West Java 16911, Indonesia.

**Keywords:** Friesian-Holstein, haptoglobin biomarker, metabolic-inflammatory disturbances, postpartum endometritis, reproductive performance, tropical dairy cows

## Abstract

**Background and Aim:**

Postpartum endometritis is a major cause of impaired fertility and economic loss in dairy production. Although metabolic, inflammatory, biochemical, and hormonal disturbances have individually been associated with uterine disease, comprehensive longitudinal evaluation of these physiological responses and their relationships with reproductive performance remains limited, particularly under tropical dairy production systems. This study aimed to longitudinally evaluate metabolic, inflammatory, biochemical, and hormonal biomarkers associated with uterine health and to determine their relationships with reproductive performance in Friesian-Holstein (FH) dairy cows with suspected and subclinical endometritis.

**Materials and Methods:**

Sixty multiparous FH dairy cows were classified by endometrial cytology into suspected endometritis (n = 30; polymorphonuclear neutrophils [PMN] 5%–<10%) and subclinical endometritis (n = 30; PMN ≥10%). Blood samples were collected on day 0 and day 30 to determine serum concentrations of non-esterified fatty acids (NEFAs), β-hydroxybutyrate (BHB), calcium (Ca), albumin, globulin, total protein (TP), haptoglobin (Hp), prostaglandin F₂α metabolite (PGFM), and cortisol. Uterine recovery was assessed by repeat cytology, while reproductive performance was evaluated using estrus intensity, service per conception (SC), and days open (DO). Statistical analyses included an independent-samples t-test, a paired-samples t-test, and a Pearson correlation analysis.

**Results:**

Cows with subclinical endometritis exhibited significantly greater uterine inflammation, higher PMN percentages, elevated NEFAs, BHB, Hp, PGFM, cortisol, globulin, and TP concentrations, and lower Ca and albumin concentrations than cows with suspected endometritis (p < 0.05). Recovery rates were lower in cows with subclinical endometritis (50.0%) than in those with suspected endometritis (83.3%). These cows also showed poorer reproductive performance, characterized by reduced estrus intensity, increased SC, and prolonged DO (p < 0.05). Among all evaluated biomarkers, Hp demonstrated the strongest associations with reproductive performance, showing a strong negative correlation with estrus intensity (r = −0.84) and strong positive correlations with SC (r = 0.84) and DO (r = 0.87) (p < 0.01). Longitudinal assessment further demonstrated partial physiological recovery between day 0 and day 30, although cows with subclinical endometritis consistently exhibited greater metabolic and inflammatory disturbances.

**Conclusion:**

Integrated longitudinal assessment demonstrated that systemic inflammation, metabolic imbalance, and endocrine alterations collectively influence uterine recovery and fertility in FH dairy cows. Hp emerged as the most informative biomarker, outperforming conventional metabolic indicators in its association with reproductive performance. These findings support incorporating Hp into biomarker-based reproductive monitoring strategies to identify cows at risk of poor fertility outcomes under tropical dairy production conditions.

## INTRODUCTION

Endometritis is one of the most prevalent postpartum uterine disorders in dairy cattle and is a major cause of impaired reproductive efficiency and economic losses. The condition has been consistently associated with reduced conception rates, increased service per conception (SC), and prolonged days open (DO), ultimately compromising herd productivity and profitability [1]. Endometritis occurs in both clinical and subclinical forms. Clinical endometritis is characterized by visible clinical signs, including purulent or mucopurulent uterine discharge, whereas subclinical endometritis is not accompanied by overt clinical manifestations, making diagnosis considerably more challenging under field conditions [2]. Consequently, accurate diagnosis of subclinical uterine inflammation relies primarily on laboratory-based techniques. Endometrial cytology, which quantifies the proportion of polymorphonuclear neutrophils (PMNs) in endometrial epithelial samples, is widely accepted as a reliable, minimally invasive method for diagnosing uterine inflammation in dairy cows [3].

Beyond localized uterine inflammation, systemic metabolic status plays a critical role in postpartum reproductive health. During the transition period, high-producing dairy cows frequently experience negative energy balance due to the substantial increase in energy demand associated with the onset of lactation, coupled with insufficient dry matter intake [4]. This metabolic challenge promotes mobilization of adipose tissue reserves, resulting in increased circulating concentrations of non-esterified fatty acids (NEFAs) and β-hydroxybutyrate (BHB), two well-established indicators of negative energy balance [5]. Elevated NEFAs and BHB concentrations have been associated with impaired immune competence, reduced neutrophil function, and increased susceptibility to postpartum uterine disorders [6]. In addition to metabolic disturbances, systemic inflammatory and endocrine responses provide valuable information regarding uterine health and reproductive status. Acute-phase proteins, particularly haptoglobin (Hp), are widely recognized biomarkers of systemic inflammation in cattle [7]. Likewise, reproductive hormones, including the prostaglandin F₂α metabolite (PGFM) and cortisol, provide important information about uterine activity, endocrine regulation, and physiological stress during the postpartum period [8]. Collectively, these physiological parameters may provide a more comprehensive understanding of the mechanisms linking uterine inflammation with reproductive performance.

Although numerous studies have independently investigated metabolic, inflammatory, biochemical, or hormonal alterations associated with postpartum uterine disorders, these physiological systems are highly interconnected and collectively influence uterine recovery and subsequent fertility. Nevertheless, most previous investigations have focused on a limited number of biomarkers or single time-point measurements, preventing comprehensive evaluation of the dynamic interactions among metabolic imbalance, systemic inflammation, endocrine responses, and reproductive performance. Furthermore, longitudinal studies integrating endometrial cytology with simultaneous assessment of metabolic, acute-phase inflammatory, biochemical, and hormonal biomarkers within the same animals remain scarce. This knowledge gap is particularly important under tropical dairy production systems, where elevated ambient temperature and humidity may exacerbate physiological stress, reduce feed intake, intensify negative energy balance, impair immune function, and adversely affect reproductive efficiency [9, 10]. In Indonesia, Friesian-Holstein dairy cows are routinely exposed to these environmental challenges, which may produce physiological responses that differ from those reported under temperate production systems [11]. Therefore, integrated longitudinal evaluation of multiple physiological biomarkers under tropical conditions is needed to improve understanding of uterine recovery and to identify clinically useful biomarkers associated with reproductive performance.

Therefore, this study aimed to longitudinally evaluate metabolic, inflammatory, biochemical, and hormonal biomarkers associated with uterine health status in Friesian-Holstein dairy cows diagnosed with suspected or subclinical endometritis using endometrial cytology. Furthermore, the study sought to determine the relationships between these physiological biomarkers, uterine recovery status, and key reproductive performance indicators, including estrus intensity, SC, and DO, under tropical Indonesian dairy production conditions. We hypothesized that cows with more severe uterine inflammation would exhibit greater metabolic imbalance, enhanced systemic inflammatory and endocrine responses, poorer uterine recovery, and inferior reproductive performance, with Hp emerging as the biomarker most strongly associated with reproductive outcomes.

## MATERIALS AND METHODS

### Ethical approval

All experimental procedures involving animals were reviewed and approved by the Ethics Committee of the Faculty of Animal Science, Hasanuddin University, Makassar, Indonesia (Approval No. 015/UN4.12/EC/VI/2025). The study was conducted in accordance with the institutional guidelines for the care and use of animals in research, the applicable national regulations governing animal experimentation in Indonesia, and the internationally accepted principles for the ethical use of animals in scientific research.

Written permission to conduct the study was obtained from the participating commercial dairy farm before animal enrolment. All procedures, including clinical examination, endometrial cytobrush sampling, and jugular venous blood collection, were performed by licensed veterinarians or trained personnel using standardized aseptic veterinary techniques to minimize stress, pain, and the risk of infection. Animals were gently restrained for the shortest possible duration, and no invasive surgical procedures were performed.

Throughout the study, all cows remained under routine commercial herd management and had unrestricted access to feed and water in accordance with normal farm practice. Animal health and welfare were continuously monitored during the sampling period, and any cow requiring veterinary treatment received appropriate clinical care immediately. No animal was deliberately harmed, euthanized, or excluded from receiving treatment for research purposes, and participation in the study did not interfere with routine herd health management or reproductive management practices.

The observational nature of the study involved procedures commonly performed during routine reproductive health examinations, and every effort was made to minimize animal discomfort while maintaining the highest standards of animal welfare throughout the investigation.

### Study period and location

The study was conducted from August 2024 to April 2025 under commercial tropical dairy production conditions in Indonesia. The study location was characterized by high ambient temperature and humidity, environmental conditions known to influence postpartum metabolic status, inflammatory responses, and reproductive performance in dairy cattle. All laboratory analyses were performed using standardized analytical procedures under controlled laboratory conditions.

### Study design

A longitudinal observational study was conducted to evaluate metabolic, inflammatory, biochemical, and hormonal parameters associated with uterine health status, uterine recovery, and reproductive performance in Friesian-Holstein (FH) dairy cows. Sixty multiparous FH dairy cows were enrolled based on postpartum uterine health status and their availability for longitudinal follow-up.

The inclusion criteria comprised multiparous FH cows with parity ≥2, clinically stable, available for sampling on day 0 (D0) and day 30 (D30), and complete reproductive records. Cows presenting severe systemic illness, concurrent clinical diseases that could markedly influence metabolic or inflammatory status, incomplete biological sampling, or incomplete reproductive records were excluded. Throughout the study, all cows were maintained under comparable housing, nutritional, and reproductive management conditions.

Based on the initial endometrial cytological findings, cows were allocated into two experimental groups: suspected endometritis (n = 30) and subclinical endometritis (n = 30). Physiological assessments and sample collections were performed at two predetermined time points, D0 and D30. D0 represented the initial postpartum uterine cytological diagnosis and baseline physiological evaluation, whereas D30 was selected to assess short-term changes in physiological status and uterine recovery.

The principal strength of the study design was the paired longitudinal assessment of metabolic, inflammatory, biochemical, and hormonal biomarkers within the same animals, allowing comprehensive evaluation of temporal physiological changes and their associations with uterine recovery and reproductive performance.

At the completion of the observation period, cows were further classified according to uterine recovery status as recovered or not recovered. Reproductive performance variables, including estrus intensity, SC, and DO, were subsequently recorded for all animals. No animals were withdrawn from the study during the experimental period.

### Diagnosis and group classification

Uterine health status was determined by endometrial cytology, evaluating the proportion of PMNs in endometrial epithelial samples, a widely accepted diagnostic method for identifying uterine inflammation in dairy cows [12]. Endometrial samples were collected using a sterile cytobrush (Minitube GmbH, Tiefenbach, Germany). The cytobrush was carefully introduced through the cervix into the uterine lumen using a protective sheath to minimize contamination during sample collection. Cellular material was immediately transferred onto clean microscope slides (Thermo Fisher Scientific, Waltham, MA, USA), air-dried, and stained using a standard cytological staining procedure as previously described [13].

Stained slides were examined using a light microscope (Olympus BX43; Olympus Corporation, Tokyo, Japan). A minimum of 200 endometrial cells were counted per sample to determine the PMN percentage, in accordance with established cytological evaluation procedures [14]. To minimize observer bias, cytological evaluations were performed by trained personnel blinded to the animals' reproductive performance records.

Based on the PMN percentage, cows were classified into two uterine health categories. Cows with PMN percentages between 5% and <10% were classified as having suspected endometritis, whereas cows with PMN percentages ≥10% were classified as having subclinical endometritis. This classification differentiated cows with mild cytological evidence of uterine inflammation from those exhibiting more severe endometrial neutrophil infiltration.

Recovery status was reassessed at D30 using repeat endometrial cytology. Cows with PMN percentages below the diagnostic threshold for subclinical endometritis were classified as recovered, whereas those with persistent PMN percentages ≥10% were classified as not recovered.

### Sample collection

Blood samples were collected from each cow at D0 and D30 to evaluate longitudinal changes in metabolic, inflammatory, biochemical, and hormonal biomarkers. To minimize physiological variation associated with circadian rhythm and feed intake, blood sampling was consistently performed during the morning before the primary feeding period.

Approximately 10 mL of blood was collected from the jugular vein using sterile disposable needles (Terumo Corporation, Tokyo, Japan) and vacuum blood collection tubes without anticoagulant (Vacutainer®, Becton, Dickinson and Company, Franklin Lakes, NJ, USA), following standard blood collection procedures for dairy cattle.

After collection, blood samples were allowed to clot at room temperature for approximately 30 min, then centrifuged at 3,000 × *g* for 15 min using a refrigerated centrifuge (Eppendorf 5810 R, Eppendorf AG, Hamburg, Germany) to obtain serum [15]. Serum was carefully transferred into sterile microcentrifuge tubes (Eppendorf AG). Samples exhibiting visible hemolysis were excluded from subsequent analyses. All serum samples were stored at −20°C until biochemical analyses were performed.

### Metabolic, biochemical, inflammatory, and hormonal analyses

Serum samples were analyzed to determine concentrations of metabolic, biochemical, inflammatory, and hormonal biomarkers associated with uterine health status. The evaluated biomarkers included NEFAs, BHB, calcium (Ca), albumin, globulin, total protein (TP), Hp, PGFM, and cortisol.

Serum NEFAs and BHB concentrations were determined using commercial enzymatic assay kits (Randox Laboratories Ltd., Crumlin, United Kingdom) according to the manufacturer's instructions and previously described methods [16]. Absorbance values were measured using a microplate reader (BioTek ELx800, BioTek Instruments Inc., Winooski, VT, USA).

Serum Ca, albumin, and TP concentrations were measured using an automated biochemical analyzer (Mindray BS-120, Mindray Bio-Medical Electronics Co., Shenzhen, China) based on standardized colorimetric methods [17]. Globulin concentration was calculated by subtracting albumin concentration from TP concentration.

Serum Hp concentrations were quantified using a commercial enzyme-linked immunosorbent assay (ELISA) kit (MyBioSource Inc., San Diego, CA, USA) according to the manufacturer's protocol. Optical density measurements were obtained using the same microplate reader, and concentrations were calculated using standard calibration curves [18].

Serum PGFM and cortisol concentrations were determined using commercial ELISA kits (MyBioSource Inc.) according to the manufacturer's instructions. Hormone concentrations were calculated from the respective standard curves generated from absorbance measurements.

All enzymatic, biochemical, and ELISA assays were performed strictly according to the manufacturers' protocols. Blank controls, calibration standards, and kit-provided quality controls were included where applicable to ensure analytical reliability. Assay sensitivity and intra- and inter-assay coefficients of variation complied with the manufacturers' specifications.

### Reproductive performance assessment

Reproductive performance was evaluated using estrus intensity, SC, and DO. Estrus intensity was assessed by visual observation of behavioral indicators, including standing heat, mounting, restlessness, increased physical activity, and vulvar mucus discharge. Estrus detection was routinely performed by trained farm personnel and independently verified by the research team to minimize observer variation. Estrus expression was scored on a three-point ordinal scale, where score 1 represented weak estrus expression, score 2 represented moderate estrus expression, and score 3 represented strong estrus expression according to previously published scoring systems [19].

Artificial insemination was performed in cows exhibiting estrus, in accordance with the farm's routine reproductive management program. The voluntary waiting period and insemination schedule followed the farm's standard reproductive protocol. Frozen semen was administered by trained artificial insemination technicians.

SC was defined as the number of inseminations required to achieve successful conception. Pregnancy diagnosis was performed by a veterinarian using the farm's routine pregnancy diagnostic procedures. DO was calculated as the interval between parturition and confirmed conception.

### Statistical analysis

Statistical analyses were performed using IBM SPSS Statistics version 26.0 (IBM Corp., Armonk, NY, USA). Data normality was evaluated using the Shapiro–Wilk test. Descriptive data are presented as mean ± standard deviation (SD).

Comparisons between cows with suspected endometritis and subclinical endometritis were performed using the independent-samples *t*-test. Changes between D0 and D30 within each experimental group were analyzed using the paired-samples *t*-test. Comparisons between recovered and not recovered cows were performed using the independent-samples *t*-test where appropriate.

Associations between physiological biomarkers and reproductive performance variables, including estrus intensity, SC, and DO, were evaluated using Pearson correlation analysis.

Data were screened for completeness and potential outliers before statistical analysis. An *a priori* sample size calculation was not performed because the study was based on an available longitudinal field dataset. Adjustment for multiple comparisons was not applied; therefore, the findings were interpreted based on statistical significance, biological plausibility, and the consistency of observed responses. Statistical significance was established at p < 0.05.

## RESULTS

### Uterine cytology and recovery status

Endometrial cytology revealed distinct differences in PMN percentages between cows with suspected endometritis and those with subclinical endometritis (Table 1). Cows with suspected endometritis had a significantly lower mean PMN percentage than cows with subclinical endometritis (7.01 ± 0.28% vs. 11.68 ± 0.52%, respectively; p < 0.05).

Based on the initial cytological evaluation, 30 cows were classified with suspected endometritis and 30 with subclinical endometritis. Among cows with suspected endometritis, 25 recovered, and 5 did not, corresponding to a recovery rate of 83.3%. In contrast, among cows with subclinical endometritis, 15 recovered and 15 did not, resulting in a recovery rate of 50.0%. The recovery rate was significantly higher in cows with suspected endometritis than in those with subclinical endometritis (p < 0.05; Table 1). These findings indicate that greater cytological evidence of uterine inflammation was associated with a lower probability of recovery during the observation period.

### Metabolic parameters

**Figure 1:**
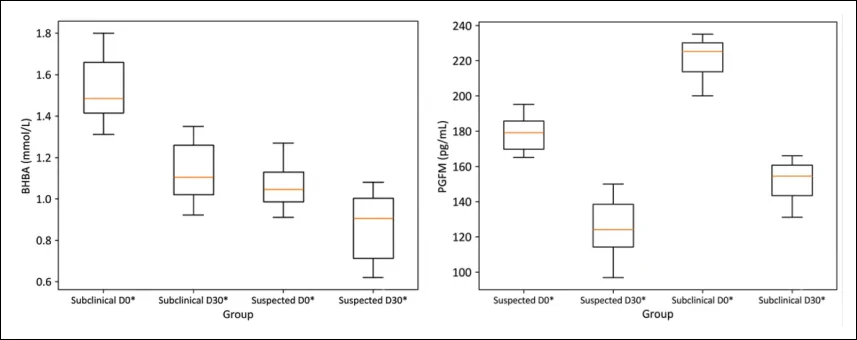
Distribution of metabolic biomarkers in dairy cows with suspected and subclinical endometritis at day 0 (D0) and day 30 (D30). (A) Non-esterified fatty acids (NEFAs). (B) β-hydroxybutyrate (BHB). Different superscripts indicate significant differences among groups (p < 0.05).

Serum concentrations of the metabolic biomarkers NEFAs and BHB at D0 and D30 are presented in Figure 1. Significant differences were observed between uterine health groups and sampling times (p < 0.05). At D0, cows with subclinical endometritis exhibited significantly higher NEFAs and BHB concentrations than cows with suspected endometritis, indicating a greater degree of metabolic imbalance (p < 0.05). In both groups, NEFAs and BHB concentrations decreased significantly from D0 to D30 (p < 0.05), suggesting partial improvement in metabolic status during the observation period.

**Table 1:** Uterine cytology-based classification and recovery status of FH dairy cows.

**Initial uterine status**	**PMN (%)**	**Total (n)**	**Recovered (n)**	**Not recovered (n)**	**Recovery rate (%)**
Suspected endometritis	7.01 ± 0.28ᵃ	30	25	5	83.3ᵃ
Subclinical endometritis	11.68 ± 0.52ᵇ	30	15	15	50.0ᵇ

Values are presented as mean ± SD, where applicable. Different superscripts (ᵃ,ᵇ) within the same column indicate significant differences between groups (p < 0.05). FH: Friesian–Holstein; PMN: Polymorphonuclear neutrophils; SD: Standard deviation.

### Biochemical and inflammatory parameters

Serum biochemical and inflammatory biomarkers are summarized in Table 2. Significant differences among uterine health groups and sampling times were observed for Ca, albumin, globulin, TP, and Hp (p < 0.05).

At D0, cows with subclinical endometritis had lower serum Ca and albumin concentrations than cows with suspected endometritis. Ca concentrations were 7.99 ± 0.18 mg/dL and 8.37 ± 0.21 mg/dL, respectively, whereas albumin concentrations were 2.95 ± 0.08 g/dL and 3.22 ± 0.11 g/dL, respectively.

Conversely, globulin, TP, and Hp concentrations were higher in cows with subclinical endometritis than in those with suspected endometritis. The highest Hp concentration was observed in cows with subclinical endometritis at D0 (1.11 ± 0.09 g/dL), followed by cows with suspected endometritis at D0 (0.84 ± 0.06 g/dL), cows with subclinical endometritis at D30 (0.62 ± 0.07 g/dL), and cows with suspected endometritis at D30 (0.43 ± 0.08 g/dL).

Between D0 and D30, Ca and albumin concentrations increased in both groups, whereas globulin and Hp concentrations decreased. Overall, cows with subclinical endometritis exhibited more pronounced biochemical and inflammatory disturbances at baseline, although partial physiological recovery was observed by D30.

**Table 2:** Serum biochemical and inflammatory parameters in FH dairy cows with suspected and subclinical endometritis at D0 and D30.

**Parameter**	**Suspected D0**	**Suspected D30**	**Subclinical D0**	**Subclinical D30**
Ca (mg/dL)	8.37 ± 0.21ᶜ	8.70 ± 0.22ᵃ	7.99 ± 0.18ᵈ	8.56 ± 0.18ᵇ
Albumin (g/dL)	3.22 ± 0.11ᵇ	3.33 ± 0.13ᵃ	2.95 ± 0.08ᵈ	3.11 ± 0.12ᶜ
Globulin (g/dL)	3.67 ± 0.15ᶜ	3.44 ± 0.17ᵈ	4.04 ± 0.17ᵃ	3.71 ± 0.14ᵇ
TP (g/dL)	6.80 ± 0.05ᶜ	6.79 ± 0.05ᵈ	7.01 ± 0.05ᵃ	6.85 ± 0.03ᵇ
Hp (g/dL)	0.84 ± 0.06ᵇ	0.43 ± 0.08ᵈ	1.11 ± 0.09ᵃ	0.62 ± 0.07ᶜ

Note: Values are presented as mean ± SD. Different superscripts (ᵃ–ᵈ) within the same row indicate significant differences among groups (p < 0.05). Ca: Calcium; D0: Day 0; D30: Day 30; Hp: Haptoglobin; SD: Standard deviation; TP: Total protein. FH: Friesian–Holstein.

### Hormonal parameters

**Figure 2:**
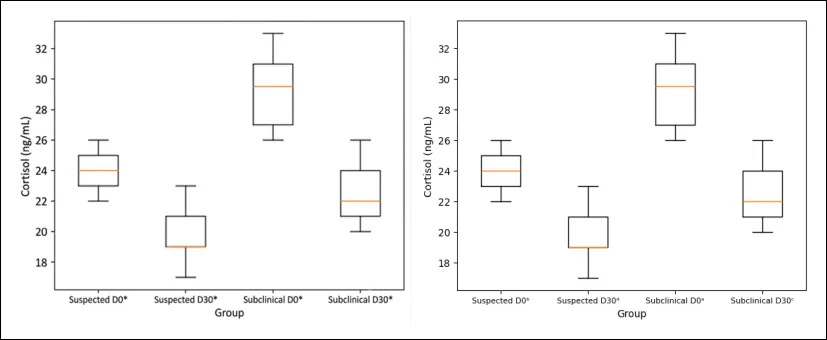
Serum concentrations of (A) prostaglandin F₂α metabolite (PGFM) and (B) cortisol in dairy cows with suspected and subclinical endometritis at day 0 (D0) and day 30 (D30). Different superscripts indicate significant differences among groups (p < 0.05).

Serum concentrations of PGFM and cortisol at D0 and D30 are presented in Figure 2. Significant differences were observed among uterine health groups and sampling times (p < 0.05).

The highest PGFM concentration was detected in cows with subclinical endometritis at D0, followed by cows with suspected endometritis at D0, cows with subclinical endometritis at D30, and cows with suspected endometritis at D30. This pattern suggests a greater prostaglandin-mediated uterine response in cows with more severe uterine inflammation during the initial assessment.

A similar trend was observed for cortisol concentrations. Cows with subclinical endometritis had significantly higher serum cortisol concentrations than cows with suspected endometritis, particularly at D0 (p < 0.05). In both groups, PGFM and cortisol concentrations decreased significantly between D0 and D30 (p < 0.05), indicating reduced endocrine activation and physiological stress during the observation period. Nevertheless, cows with subclinical endometritis consistently maintained higher hormonal concentrations than cows with suspected endometritis throughout the study.

### Reproductive performance

Reproductive performance data are presented in Table 3. Significant differences were observed between cows with suspected endometritis and those with subclinical endometritis for all evaluated reproductive variables (p < 0.05).

Cows with suspected endometritis exhibited significantly higher estrus intensity scores than cows with subclinical endometritis (2.33 ± 0.76 vs. 1.50 ± 0.51; p < 0.05). Conversely, cows with subclinical endometritis required significantly more services per conception than cows with suspected endometritis (2.50 ± 0.51 vs. 1.67 ± 0.76; p < 0.05). Likewise, DO were significantly longer in cows with subclinical endometritis than in cows with suspected endometritis (136.20 ± 23.36 vs. 104.27 ± 17.06 days; p < 0.05).

Overall, cows with subclinical endometritis exhibited poorer reproductive performance, characterized by weaker estrus expression, increased insemination requirements, and delayed conception.

**Table 3:** Reproductive performance parameters of FH dairy cows with suspected and subclinical endometritis.

**Parameter**	**Suspected endometritis**	**Subclinical endometritis**
Estrus intensity (score)	2.33 ± 0.76ᵃ	1.50 ± 0.51ᵇ
SC	1.67 ± 0.76ᵇ	2.50 ± 0.51ᵃ
DO (days)	104.27 ± 17.06ᵇ	136.20 ± 23.36ᵃ

Note: Values are presented as mean ± SD. Different superscripts (ᵃ,ᵇ) within the same row indicate significant differences between groups (p < 0.05). DO: Days open; SC: Service per conception; SD: Standard deviation. FH: Friesian–Holstein.

### Correlation between physiological biomarkers and reproductive performance

**Figure 3:**
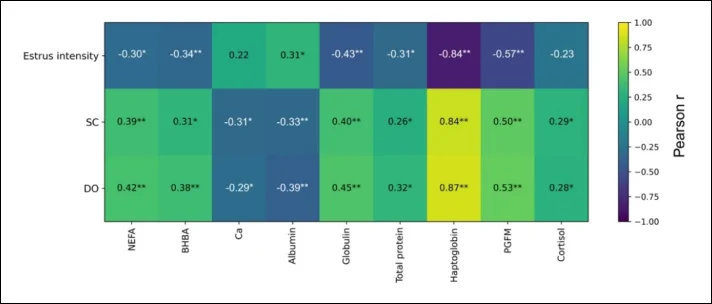
Heatmap of Pearson correlation coefficients between physiological biomarkers and reproductive performance indicators in dairy cows. Values represent correlation coefficients (r). *p < 0.05; **p < 0.01.

Pearson correlation analysis between physiological biomarkers and reproductive performance indicators is presented in Figure 3. Several metabolic, biochemical, inflammatory, and hormonal biomarkers showed significant associations with estrus intensity, SC, and DO.

Among all evaluated biomarkers, Hp demonstrated the strongest associations with reproductive performance. Hp was strongly negatively correlated with estrus intensity (r = −0.84, p < 0.01) and strongly positively correlated with SC (r = 0.84, p < 0.01) and DO (r = 0.87, p < 0.01).

Metabolic biomarkers also demonstrated significant, although comparatively weaker, associations with reproductive performance. NEFAs and BHB were negatively correlated with estrus intensity (r = −0.30 to −0.34, p < 0.05) and positively correlated with SC and DO (r = 0.31–0.42, p < 0.05).

Among the biochemical biomarkers, globulin and TP showed positive correlations with SC and DO (r = 0.26–0.45, p < 0.05), whereas albumin was positively correlated with estrus intensity (r = 0.31, p < 0.05) and negatively correlated with SC and DO (r = −0.33 to −0.39, p < 0.01).

Similarly, hormonal biomarkers were associated with reproductive outcomes. PGFM was positively correlated with SC (r = 0.50, p < 0.01) and DO (r = 0.53, p < 0.01), whereas cortisol exhibited weaker positive correlations with SC and DO (r = 0.28–0.29, p < 0.05).

Overall, the correlation analysis demonstrated that poorer reproductive performance was associated with greater metabolic stress, systemic inflammation, and endocrine activation, with Hp exhibiting the strongest association among all evaluated physiological biomarkers.

## DISCUSSION

### Overview of principal findings

The present study evaluated metabolic, inflammatory, biochemical, and hormonal parameters associated with uterine health status, recovery status, and reproductive performance in FH dairy cows. Cows with subclinical endometritis exhibited higher PMN percentages, more pronounced metabolic and inflammatory disturbances, lower recovery rates, and poorer reproductive performance than cows with suspected endometritis. Collectively, these findings indicate that greater uterine inflammation is closely associated with systemic physiological imbalance, delayed uterine recovery, and reduced reproductive efficiency.

### Uterine inflammation and recovery status

Endometrial cytology demonstrated that cows with subclinical endometritis had higher PMN percentages than cows with suspected endometritis. Increased PMN infiltration reflects an active inflammatory response within the endometrium and is widely accepted as a reliable indicator of uterine inflammation in dairy cows [20]. Persistent postpartum uterine inflammation may delay uterine involution, alter endometrial function, and reduce the ability of the uterus to support sperm transport, embryo implantation, and early embryonic development [21, 22].

In the present study, cows with suspected endometritis exhibited a higher recovery rate than cows with subclinical endometritis, suggesting that animals with less severe initial endometrial inflammation were more likely to recover during the observation period. Conversely, greater neutrophilic infiltration may indicate persistent inflammatory activity and impaired restoration of normal uterine function. These findings support the value of endometrial cytology not only as a diagnostic method but also as an indicator of recovery potential and the risk of persistent uterine inflammation.

### Metabolic imbalance and uterine health

Metabolic status differed markedly between the uterine health groups. Cows with subclinical endometritis had higher NEFAs and BHB concentrations, indicating a greater degree of negative energy balance. During early lactation, high-producing dairy cows commonly experience negative energy balance because the rapid increase in energy demand for milk production exceeds dietary energy intake [23]. This metabolic challenge promotes adipose tissue mobilization and increases circulating NEFAs and BHB concentrations.

Elevated NEFAs and BHB concentrations have been associated with impaired immune competence, reduced neutrophil function, altered inflammatory regulation, and increased susceptibility to postpartum uterine infections [24]. Consequently, cows experiencing greater metabolic stress may have a reduced capacity to eliminate uterine pathogens and resolve endometrial inflammation. The decreases in NEFAs and BHB concentrations from D0 to D30 indicate partial metabolic recovery in both groups. However, the persistently greater metabolic disturbance in cows with subclinical endometritis suggests that prolonged negative energy balance may contribute to persistent uterine inflammation and inferior reproductive performance.

### Hp as an indicator of reproductive performance

Among all evaluated physiological parameters, Hp showed the strongest associations with reproductive performance. Although NEFAs and BHB were significantly associated with SC and DO, their correlations were considerably weaker than those observed for Hp. In the present study, Hp showed the strongest negative correlation with estrus intensity and the strongest positive correlations with SC and DO. These findings suggest that systemic inflammatory status may be more closely associated with reproductive impairment than metabolic imbalance alone.

Hp is produced primarily by the liver in response to inflammatory cytokines and tissue injury and is widely recognized as a biomarker of systemic inflammation in cattle [25]. The elevated Hp concentrations observed in cows with subclinical endometritis are consistent with the greater degree of uterine inflammation identified by endometrial cytology. Moreover, the decline in Hp concentrations between D0 and D30 indicates partial resolution of the systemic inflammatory response. Nevertheless, persistently higher Hp concentrations in cows with subclinical endometritis suggest continued inflammatory activity despite partial physiological recovery. These observations highlight the potential utility of Hp in assessing reproductive prognosis in cows with endometritis.

### Potential mechanisms linking Hp and fertility

The biological relationship between elevated Hp concentrations and impaired reproductive performance likely involves complex interactions among systemic inflammation, iron homeostasis, ovarian function, and endocrine regulation. Hp binds free hemoglobin released during tissue injury, thereby reducing oxidative damage and restricting iron availability to pathogenic microorganisms. Although this mechanism is physiologically protective, sustained elevation of Hp reflects persistent systemic inflammation.

Prolonged inflammatory activity may disrupt the hypothalamic–pituitary–ovarian axis, impair follicular development and steroidogenesis, compromise luteal function, and reduce embryonic survival [26]. Inflammatory mediators may also interfere with gonadotropin responsiveness and ovarian follicular dynamics, ultimately reducing fertility. Therefore, elevated Hp concentrations may reflect an inflammatory state that contributes to weaker estrus expression, increased insemination requirements, and prolonged DO. This interpretation is consistent with the stronger associations observed for Hp than for NEFAs, BHB, and the other biochemical and hormonal parameters evaluated in this study.

### Hormonal alterations and reproductive outcomes

Hormonal parameters were also significantly associated with reproductive performance. PGFM showed positive correlations with SC and DO. Prostaglandins are key mediators of uterine inflammatory responses and play central roles in luteolysis, uterine contractility, and postpartum uterine involution [27]. Elevated PGFM concentrations may therefore reflect persistent uterine inflammation or altered prostaglandin metabolism that adversely affects reproductive function.

Cortisol also showed positive correlations with SC and DO, although these relationships were weaker than those observed for Hp. Cortisol is a principal mediator of physiological stress responses, and prolonged increases in cortisol may suppress gonadotropin secretion, impair ovarian follicular development, and reduce estrus expression [28]. The higher cortisol concentrations observed in cows with subclinical endometritis suggest that more severe uterine inflammation was accompanied by greater physiological stress. The decreases in PGFM and cortisol between D0 and D30 indicate partial normalization of endocrine responses during recovery.

### Integrated relationships among physiological parameters

The correlation analysis emphasized the close interaction among metabolic stress, systemic inflammation, endocrine regulation, and reproductive performance. NEFAs and BHB were negatively associated with estrus intensity and positively associated with SC and DO. These findings agree with previous reports demonstrating that postpartum metabolic stress impairs follicular growth, oocyte quality, steroidogenesis, and endocrine signaling, thereby reducing reproductive efficiency in dairy cattle [29].

Albumin was positively associated with estrus intensity and negatively associated with SC and DO, whereas globulin and TP showed positive associations with poorer reproductive outcomes. These relationships likely reflect the combined effects of inflammation, hepatic protein metabolism, and nutritional status. Nevertheless, the substantially stronger correlations observed for Hp suggest that persistent systemic inflammation may be a dominant physiological factor underlying impaired reproductive performance in cows with endometritis.

### Relevance under tropical dairy production conditions

The present findings are particularly relevant to dairy production under tropical Indonesian conditions. High ambient temperature and humidity may reduce dry matter intake, exacerbate negative energy balance, increase physiological stress, and compromise immune and reproductive function [9, 10]. Consequently, FH cows maintained in tropical environments may experience greater physiological challenges than those raised in temperate conditions.

Heat stress may interact with postpartum uterine inflammation by intensifying metabolic imbalance, endocrine disturbances, and systemic inflammatory responses, thereby delaying uterine recovery and impairing fertility. The present study provides contextual novelty by integrating endometrial cytology with longitudinal assessment of metabolic, inflammatory, biochemical, and hormonal parameters under tropical dairy production conditions. This comprehensive approach improves understanding of the physiological mechanisms linking uterine inflammation with reproductive performance in tropical dairy herds.

### Practical implications

From a practical perspective, these findings have important implications for precision dairy herd management. Routine monitoring of Hp may facilitate early identification of cows at increased risk of weak estrus expression, repeated insemination, delayed conception, and prolonged DO. Compared with NEFAs and BHB, Hp showed stronger associations with reproductive outcomes, suggesting that monitoring systemic inflammation may add value to reproductive management.

Furthermore, combining endometrial cytology with circulating Hp assessment may improve the identification of cows requiring intensified reproductive monitoring, nutritional intervention, or veterinary treatment. However, the present study did not establish diagnostic cutoff values or validate predictive models for Hp. Consequently, additional validation is required before its routine implementation as a cow-side decision-support biomarker.

### Strengths and limitations

A major strength of this study was the longitudinal evaluation of metabolic, inflammatory, biochemical, and hormonal parameters within the same animals at D0 and D30. Integrating endometrial cytology, uterine recovery status, and reproductive performance enabled a comprehensive assessment of the relationships between local uterine inflammation and systemic physiological responses. Conducting the study under tropical field conditions further enhanced its practical relevance to dairy production systems in Indonesia and similar environments.

Nevertheless, several limitations should be considered. The observational design precludes definitive conclusions regarding causality between physiological parameters and reproductive outcomes. Environmental, nutritional, parity-related, and management factors may also have influenced metabolic, inflammatory, hormonal, and reproductive responses. In addition, an *a priori* sample size calculation and adjustment for multiple comparisons were not performed.

Formal predictive modeling, regression-based effect-size estimation, interaction analyses between metabolic and inflammatory parameters, and machine-learning approaches were beyond the scope of the available dataset. Detailed economic analyses of reproductive losses associated with elevated Hp concentrations were also not performed because comprehensive farm-level economic data were unavailable. Furthermore, an independent validation cohort was not included, and uterine microbiome profiling, oxidative stress markers, cytokines, microRNA expression, and adipokine measurements were not investigated.

### Future research

Future investigations should include larger and more diverse dairy populations, additional longitudinal sampling points, controlled experimental designs, and independent validation cohorts. Multivariable analyses are needed to determine whether Hp independently predicts reproductive outcomes after adjustment for parity, milk yield, body condition score, nutritional status, and environmental variables.

Future studies should also establish clinically relevant Hp cutoff values, evaluate rapid field-based Hp assays, and develop predictive models integrating Hp with NEFAs, BHB, PGFM, cortisol, cytokines, oxidative stress markers, and uterine microbiome profiles. Economic analyses are additionally warranted to determine whether Hp-guided reproductive monitoring can improve herd fertility and profitability under commercial dairy production conditions.

### Summary of the discussion

The present study demonstrates that metabolic imbalance, systemic inflammation, and hormonal alterations are closely associated with uterine health status, recovery, and reproductive performance in FH dairy cows. Cows with subclinical endometritis exhibited more severe physiological disturbances, lower recovery rates, weaker estrus expression, greater SC, and prolonged DO than cows with suspected endometritis. Among all evaluated physiological parameters, Hp showed the strongest associations with estrus intensity, SC, and DO, indicating that systemic inflammation is a major physiological feature associated with reduced reproductive efficiency in cows with endometritis. These findings support further investigation of Hp as a biomarker for reproductive monitoring and management, particularly under tropical dairy production systems.

## CONCLUSION

Cows with subclinical endometritis exhibited greater uterine inflammation, as indicated by higher PMN percentages, and had a lower recovery rate than cows with suspected endometritis. This more severe uterine inflammatory status was accompanied by higher NEFAs, BHB, Hp, PGFM, and cortisol concentrations, together with lower Ca and albumin concentrations. These alterations were also reflected in poorer reproductive performance, including weaker estrus intensity, greater SC, and prolonged DO. Although several metabolic, biochemical, inflammatory, and hormonal parameters were associated with reproductive outcomes, Hp showed the strongest correlations with estrus intensity, SC, and DO.

These findings indicate that systemic inflammation is closely linked to delayed uterine recovery and reduced reproductive efficiency in FH dairy cows. The combined assessment of endometrial cytology and circulating Hp may therefore provide a useful approach for identifying cows at increased risk of persistent uterine inflammation and poor reproductive performance. Overall, Hp appears to be a promising biomarker for reproductive monitoring under tropical dairy production conditions; however, larger studies and independent validation are required before its routine application in herd-level decision-making.

## DATA AVAILABILITY

The supplementary data can be made available from the corresponding author upon request.

## GENERATIVE AI DECLARATION

The authors declare that generative artificial intelligence (AI) tools were used solely to improve language, grammar, and readability during manuscript preparation. All scientific content, data analysis, interpretation of results, and conclusions were developed and verified by the authors. The authors take full responsibility for the accuracy, integrity, and originality of the work presented, and no AI tool was listed as an author.

## AUTHORS’ CONTRIBUTIONS

HAH, YD, and ES: Conceptualization. HAH, ES, RR, AMD, and MMAR: Methodology. HAH, RR, AMD, MMAR, AASA, and MFA: Investigation. AMD, MMAR, AASA, and MFA: Data curation. HAH, YD, ES, and LP: Formal analysis. HH, AH, UA, and LP: Resources. HAH, YD, ES, LP, and HH: Supervision. HAH, AMD, and MMAR: Writing – original draft. YD, ES, RR, LP, HH, AH, UA, AASA, and MFA: Writing – review and editing. All authors have read and approved the final version of the manuscript.
